# Outbreak of Ciprofloxacin-Resistant *Shigella sonnei* Associated with Travel to Vietnam, Republic of Korea

**DOI:** 10.3201/eid2107.150363

**Published:** 2015-07

**Authors:** Jin Seok Kim, Jae Joon Kim, Soo Jin Kim, Se-Eun Jeon, Ki Yeon Seo, Jun-Kil Choi, Nan-Ok Kim, Sahyun Hong, Gyung Tae Chung, Cheon-Kwon Yoo, Young-Taek Kim, Hyeng Il Cheun, Geun-Ryang Bae, Yeong-Hee Yeo, Gang-Ja Ha, Mi-Suk Choi, Shin-Jung Kang, Junyoung Kim

**Affiliations:** Korea National Institute of Health, Cheongju-si, South Korea (J.S. Kim, S.J. Kim, S.E. Jeon, N.-O. Kim, S. Hong, G.T. Chung, C.-K. Yoo, S.-J. Kang, J. Kim);; Gyeongsangnam-do Provincial Government, Changwon-si (J.J. Kim);; Korea Centers for Disease Control and Prevention, Cheongju-si (K.Y. Seo, J.-K. Choi, Y.T. Kim, H.I. Cheun, G.-R. Bae);; Gyeongsangnam-do Public Health and Environmental Research Institute, Changwon-si (Y.-H. Yeo, G.-J. Ha);; Changnyeong Preservation of Health, Changnyeong-gun, South Korea (M.-S. Choi)

**Keywords:** CTX-M-15, ESBL, drug resistance, ciprofloxacin, cephalosporins, fluoroquinolones, antibiotic drugs, antimicrobial resistance, bacteria, Enterobacteriaceae, Shigella sonnei, shigellosis, travel-related infections, South Korea, Vietnam, enteric infections

## Abstract

We investigated an October 2014 outbreak of illness caused by *Shigella sonnei* in a daycare center in the Republic of Korea (South Korea). The outbreak strain was resistant to extended-spectrum cephalosporins and fluoroquinolones and was traced to a child who had traveled to Vietnam. Improved hygiene and infection control practices are needed for prevention of shigellosis.

*Shigella* spp. are etiologic agents of gastrointestinal disease worldwide and are frequently associated with outbreaks because of their low infectious doses and person-to-person transmission (*1*,*2*). For the treatment of persons who have severe infections, fluoroquinolones are among the first-line agents for adults; additionally, oral extended-spectrum cephalosporins are used to treat young children. However, the current emergence and spread of drug resistance in *Shigella* strains could hinder empirical antimicrobial therapy, leading to treatment failure. *S. sonnei* is the most frequently isolated species among all cases of *Shigella* infection in industrialized countries (*3*), and it has become increasingly prevalent across Southeast Asia in recent decades (*4*). Recently, international travel to areas where the disease is highly endemic has accelerated the global spread of drug-resistant *S. sonnei* to nonendemic regions. Here, we describe a travel-associated outbreak of illness caused by a *S. sonnei* strain that was resistant to extended-spectrum cephalosporins and fluoroquinolones.

## The Study

In the beginning of October 2014, six children who were vomiting and experiencing abdominal cramping and diarrhea were admitted to the local hospital in Gyeongsangnam-do, Republic of Korea (South Korea). All patients attended the same daycare center that provided care and food to children from low-income families. Fecal specimens from 6 patients were submitted to the local public health laboratory and were processed according to a standard bacterial culture method. On October 6, Korea Centers for Disease Control and Prevention was notified that *S. sonnei* phase II were identified from all fecal samples. An epidemiologic investigation was conducted to determine the extent of the outbreak and to identify the mode of transmission. A confirmed case was identified by passive and active case-finding on the basis of laboratory-identified *S. sonnei* isolates in the fecal specimens of center attendees and staff members, families of the children, and persons in the community. A probable case was defined as a person with any shigellosis symptoms and an epidemiologic link to infected patients whose cultures were negative. The children’s guardians were interviewed by using a standardized questionnaire that requested information on symptoms, food consumption, recent travel history, and contact persons. This investigation was part of a public health emergency response and was accordingly exempt from institutional review board approval.

The investigation revealed that an 8-year-old boy (the index case-patient in this outbreak) had recently returned after visiting family in Vietnam, where *S. sonnei* infection is highly endemic. He had experienced sustained diarrheal episodes since his return, and after returning to the daycare center, children in the daycare center began having similar symptoms. Cases of shigellosis were also identified among the grandparents of the index case-patient and a person the family visited in a geographically distant location on September 27. No isolates were obtained from the environmental samples collected, including foods, drinking water, and surface swab specimens of the daycare facility.

**Figure 1:**
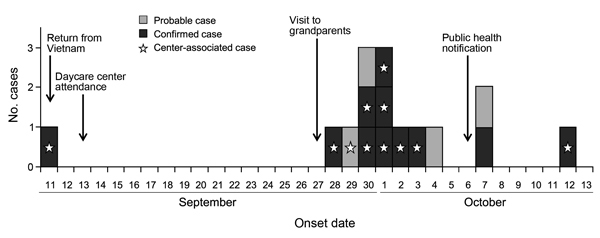
Epidemic curve of the outbreak of illness caused by *Shigella sonnei* infection, by symptom onset date, South Korea, 2014. Black bar sections indicate laboratory-confirmed cases; white bar sections indicate probable cases; stars indicate cases found in daycare center. Arrows indicate dates of the events for an index case-patient with travel history to Vietnam and of public health notification of the outbreak.

Eleven laboratory confirmed and 4 probable cases were identified during this outbreak. The median age of the patients in the daycare center was 7.8 (range 4–13) years. Overall, the reported symptoms were diarrhea (≥3 loose stools during 24 hours) and abdominal cramping; 4 patients were asymptomatic but their stool samples were culture-positive. Of the 15 persons who became ill (Figure 1), 10 were treated with cefotaxime or ciprofloxacin, after which their stool samples were culture-negative. For 5 patients with continuing positive fecal culture, antibiotic drug treatment was later changed to cabapenems (meropenem or imipenem). According to local infection control guidelines, symptomatic patients were isolated in single-bed rooms until 2 consecutive fecal cultures tested negative for *S. sonnei*. To prevent the further spread of the disease, public health interventions were encouraged during the outbreak period: enforced handwashing at predetermined times at the daycare facility, strict hygiene measures in affected households, education about shigellosis, and environmental disinfection of the facility.

**Figure 2:**
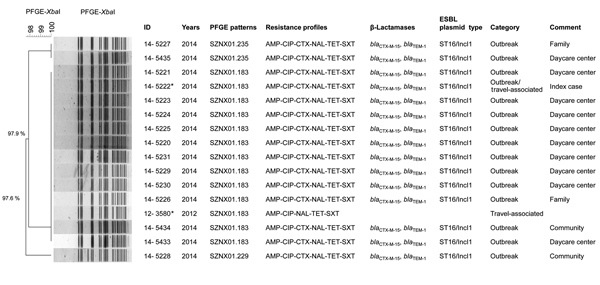
*Xba*I pulsed-field gel electrophoresis patterns of *Shigella sonnei* strains identified during a 2014 outbreak in South Korea and 2 isolated from samples from persons in Vietnam. The dendrogram was constructed by using Dice coefficient and UPGMA clustering, with 1.5% optimization and 1.5% position tolerance. Antibiotic resistance profiles and resistance determinants to extended-spectrum cephalosporins and fluoroquinolones are plotted next to the dendrogram. All strains had QRDR mutations GyrA(S83L,D87G) and ParC(S80I). *Strains 14-5222 and 12-3580 originated in Vietnam. AMP, ampicillin; CIP, ciprofloxacin; CTX, cefotaxime; NAL, nalidixic acid; TET, tetracycline; SXT, trimethoprim/sulfamethoxazole; QRDR, quinolone resistance–determining region. Scale bar indicates percentage relatedness.

Laboratory-confirmed strains of *S. sonnei* were sent to Korea National Institute of Health for further characterization. All 15 isolates had identical or highly similar pulsed-field gel electrophoresis (PFGE) patterns after the *Xba*I digestion of chromosomal DNA. The main PFGE pattern of this outbreak (SZNX01.183; PFGE pattern number assigned by Korea National Institute of Health) had not been previously reported in domestic cases, and the isolate was genetically indistinguishable from a ciprofloxacin-resistant *S. sonnei* strain isolated from a traveler returning from Vietnam during 2012 (Figure 2).

On the basis of MICs of antimicrobial agents determined by using a broth microdilution method (*5*), the outbreak strains were found to be resistant to both extended-spectrum cephalosporins (cefotaxime, MIC >32 μg/mL) and fluoroquinolones (ciprofloxacin, MIC >8 μg/mL). The strains were also resistant to tetracycline and trimethoprim/sulfamethoxazole but were susceptible to chloramphenicol, gentamicin, amikacin, and carbapenem. For azithromycin, an alternative oral agent for shigellosis, MICs were 1–2 μg/mL (Table). Extended-spectrum β-lactamase (ESBL) typing by using PCR and further sequencing (*6*) showed that all isolates carried the *bla*_CTX-M-15_ and *bla*_TEM-1_ genes. ESBL plasmid of *S. sonnei* isolate from the index case-patient were successfully transferred to the recipient *E. coli* J53 Azi^R^ strain. PCR-based *inc/rep* typing and pMLST analysis of a transconjugant strain (*7*,*8*) showed that this ESBL plasmid was of the ST16/IncI1 type, which was previously identified in strain pKHSB1 from Vietnam (*9*). The genetic environment of the *bla*_CTX-M-15_ gene was analyzed by PCR and sequencing with specific primers for the insertion sequences IS*Ecp1* and *orf477* (*6*). An intact IS*Ecp1* and truncated *orf477* were identified at 48 bp upstream and downstream of the *bla* gene, which has also been found in CTX-M-15-encoding plasmids from *Enterobacteriaceae* (*6*,*9*,*10*).

**Table:** Susceptibility profiles of outbreak *Shigella sonnei* isolate from index case-patient and *Escherichia coli* transconjugant strain used for testing, South Korea, 2014

Antimicrobial agent(s)	MIC, μg/mL		
	*Shigella sonnei* 14-5222	*E. coli* J53	*E. coli* J53, TC-14-5222
Nalidixic acid	>128	4	4
Ciprofloxacin	8	<0.12	<0.12
Ampicillin	>64	>64	>64
Ampicillin/sulbactam	16	4	16
Amoxicillin/clavulanate	16	8	16
Cefoxitin	4	4	4
Ceftazidime	8	<0.25	8
Cefotaxime	64	<0.25	64
Cefotaxime/clavulanate	<0.12	<0.12	<0.12
Cefepime	4	<1	4
Ceftriaxone	128	<0.12	128
Cefpodoxime	>32	0.5	>32
Cephalothin	>64	32	>64
Meropenem	<1	<1	<1
Imipenem	<2	<2	<2
Piperacillin/tazobactam	<4	<4	<4
Streptomycin	>128	4	4
Tetracycline	128	<2	<2
Chloramphenicol	8	8	8
Trimethoprim/sulfamethoxazole	>16	<1	<1
Gentamicin	2	<1	<1
Amikacin	<4	<4	<4
Azithromycin	1	1	1

The outbreak strains had 2 mutations in the quinolone resistance-determining region of *gyrA* (Ser83Leu and Asp87Gly) and 1 mutation in *parC* (Ser80Ile [Figure 2]), which have been reported to be responsible for ciprofloxacin resistance in *S. sonnei* (*11*). However, *gyrB* and *parE* mutations and plasmid-mediated quinolone resistance genes were not detected (*12*).

## Conclusions

We describe a shigellosis outbreak affecting children attending a daycare center, their family members, and residents of the surrounding community. To limit the extent of the outbreak, laboratory investigations of outbreak strains and infection-control measures including contact isolation and hand hygiene were immediately implemented, which may have contributed to preventing the further spread of this multidrug-resistant *S. sonnei* strain.

The outbreak strain was resistant to extended-spectrum cephalosporins and fluoroquinoloness and was introduced by a daycare center attendee who had returned from travel to Vietnam. The *bla*_CTX-M-15_ gene in *S. sonnei* was first described in 2005 (*13*) and since then has been reported worldwide; we described an outbreak of CTX-M-15–producing *S. sonnei* in Korea in 2008 (*6*). The PFGE pattern of the 2008 outbreak strain (SZNX01.176) showed only 82.8% genetic similarity with that of the outbreak strains of the current study but was observed in several traveler-associated cases originating from China. These findings suggest that, despite the lack of direct evidence, various antimicrobial drug–resistant *S. sonnei* clones have been imported across geographic regions and may eventually spread globally and lead to increased illness and death rates.

In summary, we report a shigellosis outbreak in South Korea caused by a ciprofloxacin-resistant CTX-M-15–producing *S. sonnei* strain that originated from Vietnam. Because international travel can contribute to the spread of multidrug-resistant pathogens, enhanced surveillance is necessary to control the dissemination of antimicrobial drug resistance. Improved hygiene, infection control plans, and better education for travelers are also required.
